# Typical Diagnostic Reference Levels of Radiation Exposure on Neonates Under 1 kg in Mobile Chest Imaging in Incubators

**DOI:** 10.3390/jimaging11030074

**Published:** 2025-02-28

**Authors:** Ioannis Antonakos, Matina Patsioti, Maria-Eleni Zachou, George Christopoulos, Efstathios P. Efstathopoulos

**Affiliations:** 2nd Department of Radiology, National and Kapodistrian University of Athens, 1st Rimini Str., Chaidari Athens, 12461 Attica, Greece; matpatsioti@med.uoa.gr (M.P.); zachoumar@med.uoa.gr (M.-E.Z.);

**Keywords:** neonatal radiation dose, diagnostic reference level (DRL), typical values, radiation protection

## Abstract

The purpose of this study is to determine the typical diagnostic reference levels (DRLs) of radiation exposure values for chest radiographs in neonates (<1 kg) in mobile imaging at a University Hospital in Greece and compare these values with the existing DRL values from the literature. Patient and dosimetry data, including age, sex, weight, tube voltage (kV), tube current (mA), exposure time (s), exposure index of a digital detector (S), and dose area product (DAP) were obtained from a total of 80 chest radiography examinations performed on neonates (<1 kg and <30 days old). All examinations were performed in a single X-ray system, and all data (demographic and dosimetry data) were collected from the PACS of the hospital. Typical radiation exposure values were determined as the median value of DAP and ESD distribution. Afterward, these typical values were compared with DRL values from other countries. Three radiologists reviewed the images to evaluate image quality for dose optimization in neonatal chest radiography. From all examinations, the mean value and standard deviation of DAP was 0.13 ± 0.11 dGy·cm^2^ (range: 0.01–0.46 dGy·cm^2^), and ESD was measured at 11.55 ± 4.96 μGy (range: 4.01–30.4 μGy). The typical values in terms of DAP and ESD were estimated to be 0.08 dGy·cm^2^ and 9.87 μGy, respectively. The results show that the DAP value decreases as the exposure index increases. This study’s typical values were lower than the DRLs reported in the literature because our population had lower weight and age. From the subjective evaluation of image quality, it was revealed that the vast majority of radiographs (over 80%) met the criteria for being diagnostic as they received an excellent rating in terms of noise levels, contrast, and sharpness. This study contributes to the recording of typical dose values in a sensitive and rare category of patients (neonates weighing <1 kg) as well as information on the image quality of chest X-rays that were performed in this group.

## 1. Introduction

Newborns, during their hospitalization at the Neonatal Intensive Care Unit (NICU), undergo several chest X-ray examinations for the diagnosis and evaluation of serious medical problems by the attending physician [1]. Respiratory distress syndrome (RDS), infections, patent ductus arteriosus, chronic lung disease, transient tachypnea of the newborn (TTN), and meconium aspiration syndromes are some of the various lung problems to which premature babies are more prone. Regular chest X-ray imaging in newborns in these units is also essential for the verification of catheter positioning within their bodies [2]. These examinations are performed using mobile X-ray equipment. The positioning of the system in the NICU differs from traditional radiography. The mobile X-ray machine is placed around the incubator and other life-saving equipment, making the examination particularly challenging [3].

Pediatric patients, especially neonates, require special attention to radiation exposure since they are more radiosensitive than adults and have a longer life expectancy, which increases the probability of developing stochastic effects of radiation, such as cancer [3]. Also, due to the small size of their bodies, several organs of newborns are located within the primary beam of radiation and are exposed to it. In fact, the risk of cancer per unit dose is estimated to be 2–3 times greater in pediatric patients than in adults [4]. Therefore, it is important to ensure the principles of radiation protection while maintaining image quality.

To control and optimize radiation exposure during radiological examinations, the International Commission on Radiological Protection (ICRP) and the European Commission recommended diagnostic reference levels (DRLs) [5]. The ICRP publication 73 in 1996 introduced DRLs as “a form of investigation level” that “will be intended for use as a simple test for identifying situations where levels of patient dose or administered activity are unusually high” [4]. The ICRP publication 135 in 2017 added that DRLs are “an effective tool that aids in optimization of protection in the medical exposure of patients for diagnostic and interventional procedures” [6]. The DRLs are not dose limits and can be exceeded if it is clinically necessary. They are defined for patient groups, not for individual patients, and can be categorized into regional, national, or local [7].

According to the European Commission (Radiation Protection No. 185), “a local DRL (LDRL) is based on the 3rd quartile (the 75th percentile) value of the distribution of patient doses obtained from radiology departments in a single large healthcare facility or a group of healthcare facilities, for a defined clinical imaging task (i.e., common indication-based protocol) surveyed for standardized patient groupings” [8]. For a single X-ray system, the ICRP publication 135 in 2017 provides recommendations for the use of “typical value”. Typical value is defined as “the median value of the distribution, as there are insufficient data to use the third quartile” [6]. Dosimetry parameters that are commonly used to determine DRLs in radiology are the dose-area product (DAP) and entrance surface dose (ESD) [9]. After collecting exposure data and calculating the median dose, these values are compared with the DRL values. If these values do not exceed the local DRL values, the test is repeated after three years. However, if these values are higher than local DRL values, then recommendations for optimization strategy are necessary [6].

According to European Guidelines on DRLs for pediatric imaging [9], the classification of patients is a crucial factor for establishing DRLs. The size of pediatric patients ranges from premature babies (e.g., 300–400 g) to obese adolescents (> 80 kg body weight). For all body examinations, the grouping of patients recommended in this guideline is based on the parameter weight and age [Table 1]. The first weight group (neonate, <5 kg) applies to newborn babies but excludes those in incubators, as this weight category does not represent this patient subgroup.

Despite the recommendations and the importance of establishing DRLs for this specific patient group (newborns in NICU), the majority of studies identifying DRLs primarily focus on adult populations. This emphasis on the adult population is explained by the ease of data collection in this group due to the greater number of tests and the smaller dose range depending on their age and weight compared to pediatric patients [5,7,8,9,10,11,12,13,14]. However, pediatric studies, especially those focusing on newborns weighing less than 1 kg, are limited despite the high risks associated with radiation exposure and the need for DRLs. To our knowledge, only a few studies have focused on this area [15,16,17].

This study aims to determine typical radiation exposure values for chest radiographs in neonates (<1 kg) in mobile imaging at a University Hospital in Greece and to compare these values with the existing DRL values from the literature to optimize imaging protocols.

## 2. Material and Methods

### 2.1. Patient Study

**Figure 1:**
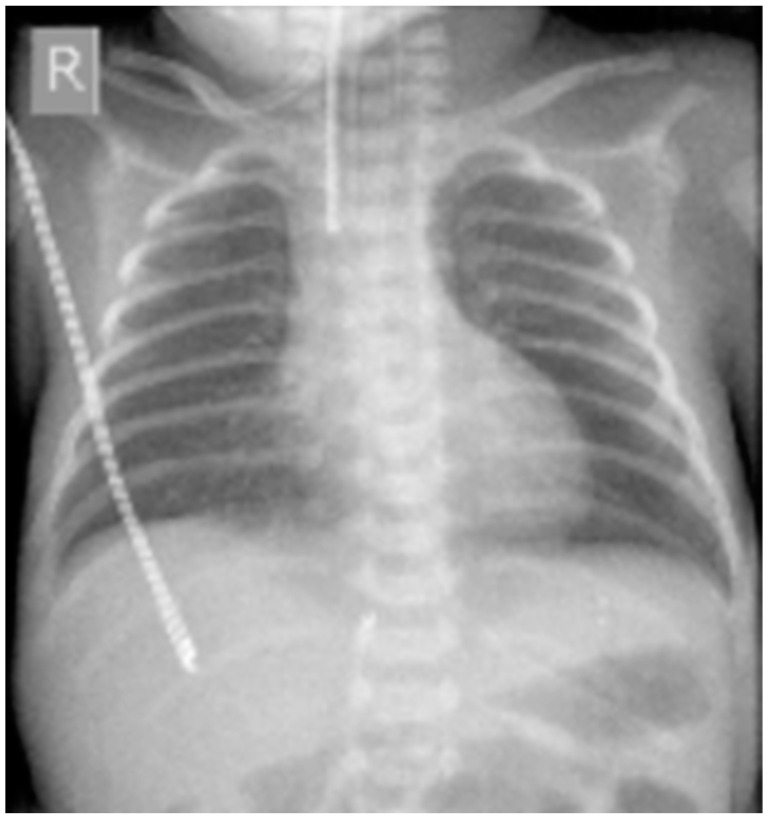
Neonatal chest X-ray.

Our retrospective study consisted of 30 patients and was conducted at a University Hospital in Greece between June 2024 and November 2024. All patients were newborns who were hospitalized in the neonatal intensive care unit (NICU), with serious medical illnesses and weighed less than 1 kg. A total of 80 chest radiography examinations performed on neonates of both genders were included in this study [Figure 1].

### 2.2. Data Acquisition

All chest X-ray examinations were performed at the mobile X-ray unit FDR nano FUJIFILM with the newborns in the incubator and the flat panel in a tray. The exposure parameters were set manually by the radiographers. For each examination, patient data, such as sex, age, and weight, and the exposure parameters, such as the applied tube voltage (kV), tube current (mA), exposure time (s), field of view (FOV), and exposure index of the digital detector (S), were collected from Paxera Ultima. The S value is an exposure index that measures the signal intensity generated by a digital detector for a specific examination. This exposure index is determined by the remnant radiation, which includes the primary radiation that passes through the patient and the scattered radiation emitted from the patient. Then, the remnant radiation is absorbed by a digital detector and is converted to electronic signals. Therefore, the exposure index does not directly correspond to the patient’s radiation dose and is relative to the image quality. For each digital detector system, there are different methods that estimate the exposure index. According to the manufacturer, the exposure index for the mobile X-ray unit FDR nano FUJIFILM is symbolized by the letter S and is inversely related to the exposure (200/S ∝X(mR)) [18].

The dose area product (DAP) values were measured by a calibrated DAP meter. The entrance skin dose (ESD) values were calculated according to the following formula:

(1) $$ESD=Output\cdot {\left(\frac{kVp}{80}\right)}^{2}\cdot mAs\cdot {\left(\frac{100}{FFD}\right)}^{2}\cdot BSF$$

where output is Air kerma per mAs in mGy/mAs, FFD is the focal-film distance in cm, and BSF is the backscatter factor. For our study, the FFD was 100 cm, and the BSF was 1.1 [19,20,21].

During the data analysis, the typical values for chest X-ray examination in neonates (<1 kg) were calculated as the median value of DAP and ESD distribution. Then, these typical values were compared with the existing DRL values from the literature. These typical values could then be used as a guideline, similar to local DRLs, in order to check if further optimization in this facility is needed. Three radiologists, with experience in chest radiography in neonates, evaluated the image noise, sharpness, contrast, and diagnostic confidence for the subjective assessment of the image quality. The evaluation was based on the Likert scale, a five-point scale [22] in which value 1 represents a perfect diagnostic image, and the value 5 is a non-diagnostic image [Table 2].

### 2.3. Statistical Analysis

In this study, SPSS^®^ v. 21.0 statistical software (SPSS Inc., Chicago, IL, USA) was used for statistical analysis. For all studies, a *p*-value ≤ 0.05 corresponded to a statistical significance. A test of normality was performed for the variables of age, DAP, and ESD, and according to the Kolmogorov–Smirnov test, the assumption of normality was not satisfied for any variable. To determine whether there was a statistically significant difference in the mean value of DAP between sex and age, the Independent Samples *t*-test and Spearman correlation were applied.

## 3. Results

### 3.1. Patient Population

Among the 30 newborns [Appendix A], the minimum number of examinations performed was one per newborn, while the maximum was 13 chest X-rays per newborn. The age of the total patient population ranged from 0 to 30 days, and the average age was 7.05 days. A total of 71.3% of newborns were female, with a mean age of 8.6 days, while 28.8% were male, with a mean age of 2.65 days. There was no statistical difference between the mean value of DAP in terms of the two genders (*p* = 0.553) and age (*p* = 0.138).

### 3.2. Radiation Exposure Parameters

Regarding the mean value and standard deviation of the scanning parameters, X-ray tube voltage was 51.1 ± 2.9 kV (range: 42.0–59.0 kV), tube current was 30.2 ± 1.6 mA (range: 25–34), exposure time was 38.3 ± 18.4 ms (range: 19.0–66.0 ms), mAs was calculated to be 1.2 ± 0.6 mAs (range: 0.6–2.0 mAs), and the S value was 651.1 ± 358.8 (range: 121–1630) [Table 3].

Regarding the exposure parameters, the mean value and standard deviation of DAP was 0.13 ± 0.11 dGy·cm^2^ (range: 0.01–0.46 dGy·cm^2^), and ESD was measured as 11.55 ± 4.96 μGy (range: 4.01–30.4 μGy). The typical values are represented as the median value of DAP (0.08 dGy·cm^2^) and ESD (9.87 μGy) [Table 4].

**Figure 2:**
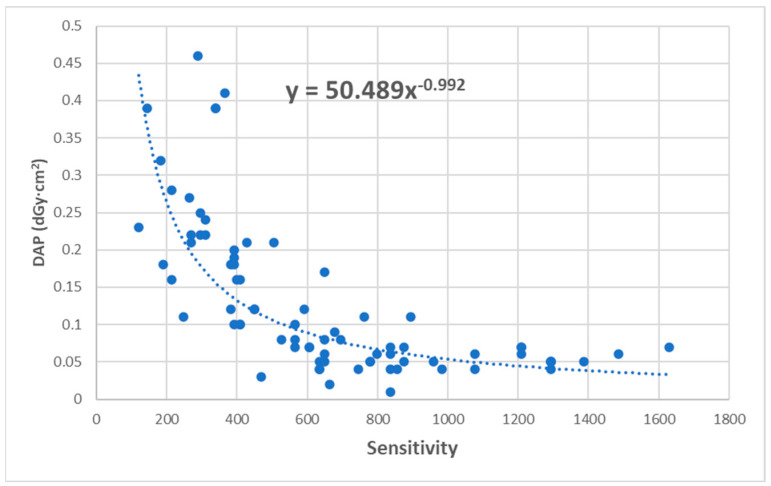
Distribution of dose area product (DAP) as a function of S index.

The DAP values concerning the S index are shown in Figure 2. From the graph, it can be observed that as the S index increases, the DAP value decreases exponentially, as recommended by the manufacturer. The equation of the trendline is given by:

(2) $$DAP=50.489\cdot S^{0.992}$$

### 3.3. Subjective Image Quality Evaluation

**Figure 3:**
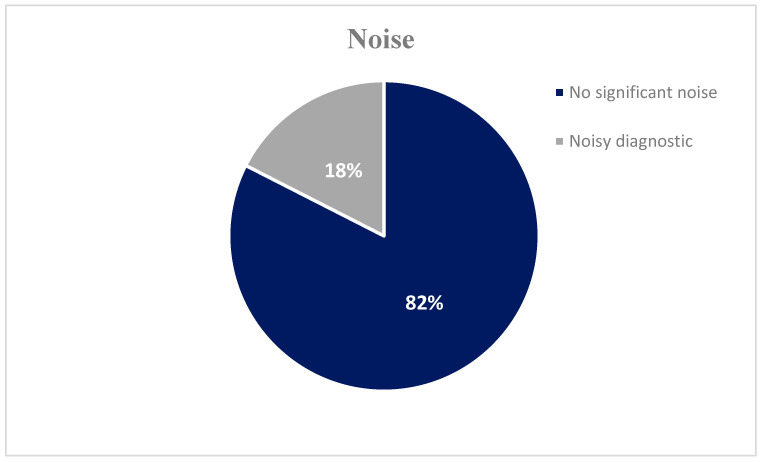
Subjective image quality assessment scores: 82% of the X-ray images were evaluated as having no significant noise, while 18% were assessed with a noisy diagnostic evaluation.

**Figure 4:**
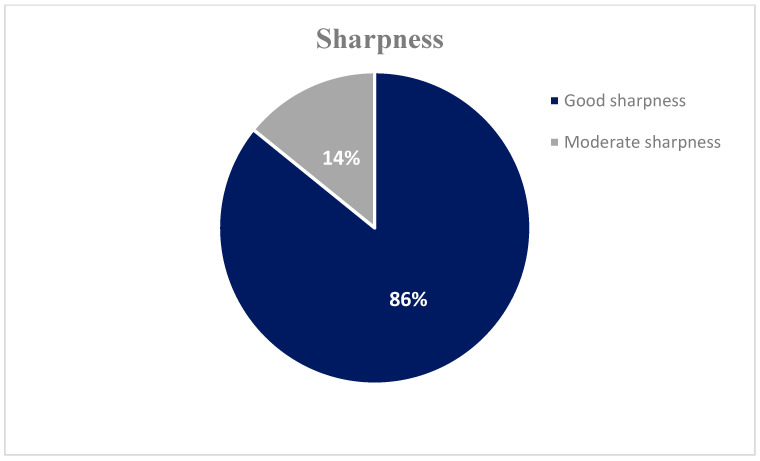
Subjective image quality assessment scores: 86% of the X-ray images were evaluated as having good sharpness, while 14% were assessed as having moderate sharpness.

**Figure 5:**
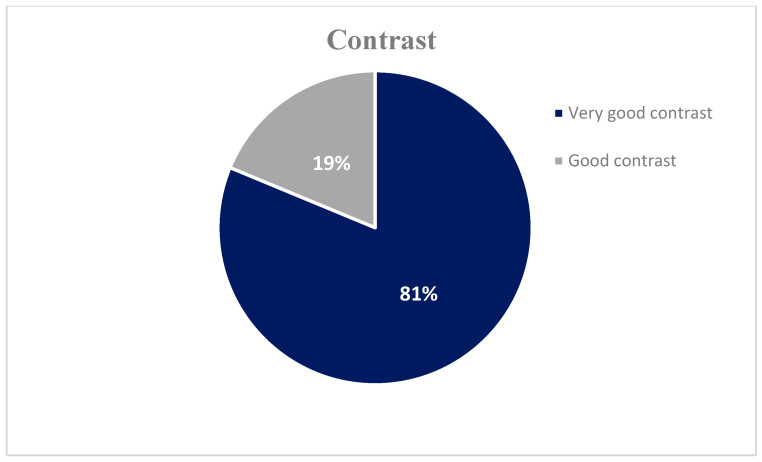
Subjective image quality assessment scores: 81% of the X-ray images were evaluated as having very good contrast, while 19% were assessed as having good contrast.

The results of the subjective image quality assessment scores are presented in Table 5 and Figure 3, Figure 4 and Figure 5. None of the images were evaluated as non-diagnostic images. All subjective parameters (noise, sharpness, and contrast) were rated by all three radiologists as a grade 2 (no significant noise, good sharpness, and very good contrast) or 3 (noisy diagnostic, moderate sharpness, and good contrast).

## 4. Discussion

In this study, we calculated the radiation exposure of neonates who underwent chest X-ray examinations to determine the typical values and then compared them with the DRL values available in the literature to assess whether protocol optimization is required. Neonates often require repeated chest X-ray examinations due to their unique physiological vulnerabilities and the complexity of their medical conditions, such as respiratory distress syndrome (RDS), pneumothorax, congenital heart defects (CHD), and bronchopulmonary dysplasia (BPD). The benefits of these examinations are important for their survival compared to the stochastic effects of radiation that may arise in the future. All the patients included in this study were premature neonates aged less than 30 days (mean age of 8.6 days) and weighing less than 1 kg. Therefore, their size was significantly smaller than that of newborns (~3 kg) and pediatric patients (5–80 kg). Consequently, the average DAP and ESD values obtained from the chest X-rays were low (0.13 ± 0.11 dGy·cm^2^ and 11.55 ± 4.96 μGy) [Table 4].

In this study, the typical values for the exposure parameters DAP and ESD were calculated, as determined in the ICRP publication 135 from 2017 for a single X-ray system [6]. The typical values for DAP and ESD in our study (0.08 dGy·cm^2^ and 9.87 μGy) are lower compared to all DRL values available in the literature [Table 6].

The exceptions are the DRL values that are reported in A. Schegerer et al.’s publication [14] (0.03 dGy·cm^2^), R. Gilley et al.’s publication [16] (0.03 dGy·cm^2^), and T.J.M. Minkels et al.’s publication [17] (0.02 dGy·cm^2^), in which the DRLs were lower than ours (0.08 dGy·cm^2^). These differences are because the DRL values from the literature are based on the 3rd quartile (the 75th percentile) of the DAP and ESD distribution, while the typical values in this study are based on the median value of the exposure parameters’ distribution [6]. Additionally, the classification of patients was carried out using different methods in each study. In our study, the patients belonged to the weight category <1 kg and age <30 days, while the DRL values in the literature were for pediatric patients: 6 out of the 11 studies in the literature [8,12,14,15,16,17] included populations in the weight category <5 kg, while the other 5 studies [5,7,9,10,13] classified patients based on age (<1 year).

Based on the trendline in Figure 2, we conclude that the DAP and S index parameters are inversely proportional. This conclusion aligns with the manufacturer’s relationship, where the exposure index for the mobile X-ray unit FDR Nano FUJIFILM is inversely related to the exposure [18].

The findings from the objective image evaluation reveal a high degree of diagnostically acceptable images with excellent noise (82%), sharpness (86%), and contrast (81%) characteristics and only a small percentage (14–19%) of moderate quality but diagnostic images. This, combined with the low radiation doses, provides confidence to those involved in performing the correct procedure; however, there is room for further optimization of the imaging. Certain techniques commonly used to obtain diagnostic-quality images while minimizing radiation exposure, such as shielding sensitive neonatal organs, appropriate positioning of neonates, and equipment settings, should be reviewed regularly.

This study has some limitations. Firstly, although radiation risk is higher in neonates and the determination of DRLs in premature neonates is necessary, there were not enough studies in the literature focusing on patients weighing <1 kg and aged <30 days old. Therefore, it was impossible to compare our results with corresponding findings from the literature. Secondly, the 80 chest X-ray examinations represent a sample from a relatively small population group (30 patients). Although the European Commission (Radiation Protection No. 185) [8] recommends that a representative sample of at least 10 patients is ample to determine the DRLs for each institute, a larger population group may be necessary for this specific patient group, which is an area that has not been investigated in depth. Additionally, the chest X-rays were performed using a mobile X-ray system, where the FFD may not have been consistent across all examinations, which could have resulted in variations in the ESD (entrance surface dose) parameter.

## 5. Conclusions

Establishing diagnostic reference levels (DRLs) is a critical step in optimizing medical imaging procedures and ensuring patient safety, especially in sensitive populations such as neonates. The recording of these values, in combination with the assessment of the image quality, is a decisive tool for the optimization of imaging techniques and can be considered a reference point, allowing facilities to compare their practices against national or international standards.

## Figures and Tables

**Table 1:** Patient grouping for pediatric DRLs, as recommended by the European Guidelines [9].

Description	Weight Group	Age Group Based on Weight-for-Age Charts	Most Common Age Groups Used for the NDRLs (or Equivalent)
Neonate	<5 kg	<1 m	0 y
Infant, toddler, and early childhood	5–<15 kg	1 m–<4 y	1 y
Middle childhood	15–<30 kg	4–<10 y	5 y
Early adolescence	30–<50 kg	10–<14 y	10 y
Late adolescence	50–<80 kg	14–<18 y	15 y

**Table 2:** Lickert scale for subjective image quality evaluation.

Image Characteristics	1	2	3	4	5
Noise	Minimal or no noise	No significant noise	Noisy diagnostic	Significant noise—affects diagnosis	High-level noise—non-diagnostic
Sharpness	Excellent sharpness	Good sharpness	Moderate sharpness	Poor sharpness—bad visibility	Zero-visibility—non-diagnostic
Contrast	Excellent contrast	Very good contrast	Good contrast	Poor contrast—unsatisfactory visualization	Non-diagnostic—image similar to the use of no contrast

**Table 3:** Mean values and standard deviation of scanning parameters for chest radiography of neonates.

Scanning Parameters	
Tube voltage (kVp)	51.1 ± 2.9
mAs	1.2 ± 0.6
Sensitivity index of digital detector (S)	651.1 ± 358.8
FFD (cm)	100

**Table 4:** Minimum, maximum, mean value, standard deviation (st.d), and median value for DAP (dGy·cm^2^) and ESD (μGy). Τhe median value of DAP and ESD represents the typical values.

Radiation Exposure Quantities				
	Minimum	Maximum	Mean ± st.d	Median
DAP (dGy·cm^2^)	0.01	0.46	0.13 ± 0.11	0.08
ESD (μGy)	4.01	30.14	11.55 ± 4.96	9.87

**Table 5:** Subjective image quality evaluation. Mean value and standard deviation of the score of the 3 radiologists for the parameters noise, sharpness, and contrast for 80 chest X-ray examinations.

Subjective Image Quality Evaluation				
	Radiologist 1	Radiologist 2	Radiologist 3	Total
NOISE	2.2 ± 0.4	2.2 ± 0.4	2.2 ± 0.4	2.2 ± 0.4
SHARPNESS	2.1 ± 0.3	2.2 ± 0.4	2.1 ± 0.3	2.1 ± 0.3
CONTRAST	2.2 ± 0.4	2.2 ± 0.4	2.2 ± 0.4	2.2 ± 0.4

**Table 6:** Comparison of typical values (median value of DAP and ESD) with literature.

Reference	Age/Weight Category	DAP [dGy·cm^2^]	ESD [μGy]
This study	<1 kg	0.08	9.87
R. Gilley et al. [16]	<1 kg	0.03	-
T.J.M. Minkels et al. [17]	600–1000 g	0.02	-
K. Alzyoud et al. [9]	0–1 y	-	130
L. Hora et al. [12]	<5 kg	0.09	-
G. Compagnone [13]	0 y	0.14	-
National Diagnostic Reference Levels in Japan (2020) [7]	0–1 y	-	200
A. Schegerer et al. [14]	<3 kg	0.03	-
RADIATION PROTECTION N° 185 [8]	<5 kg	0.15	-
B. Mohsenzadeh et al. [5]	<1 y	-	60
A. Bouaoun et al. [15]	<4 kg	0.23	55.2
H. Kim et al. [10]	0 y	0.5	-

## Data Availability

Data are contained within this article.

## Appendix A

**Table A1:** Patients’ characteristics, scanning parameters, and radiation exposure quantities for each chest X-ray examination.

No.	Sex	Age (d)	kVp	mA	Exposure Time (ms)	Sensitivity	Target Exp. Ind.	DAP (dGy∙m^2^)	mAs	Output	ESD (μGy)
1	M	3	51	30	27	960	876	0.04	0.8	22.9	12.93
2	F	11	53	30	53	241	876	0.37	1.6	25.1	30.14
3	F	15	55	34	19	649	876	0.06	0.6	27.4	14.36
4	F	15	52	30	21	1077	876	0.06	0.6	23.8	6.97
5	F	14	52	30	21	1294	876	0.05	0.6	23.8	6.97
6	F	15	49	30	66	504	876	0.21	2.0	20.4	16.63
7	F	16	52	30	21	1208	876	0.07	0.6	23.8	6.97
8	F	17	49	30	33	1208	876	0.07	1.0	20.4	8.31
9	F	18	52	30	21	1294	876	0.05	0.6	23.8	6.97
10	F	19	49	30	66	400	876	0.16	2.0	20.4	16.63
11	F	20	52	30	21	1208	876	0.06	0.6	23.8	6.97
12	F	21	55	34	21	650	279	0.17	0.7	21.7	8.05
13	F	23	52	30	21	1387	876	0.05	0.6	23.8	6.97
14	M	5	44	25	32	1630	876	0.07	0.8	15.1	4.01
15	M	6	53	30	27	391	876	0.19	0.8	25.1	9.83
16	M	7	49	30	66	527	876	0.08	2.0	20.4	16.63
17	F	0	56	34	24	565	876	0.07	0.8	28.3	12.46
18	F	1	49	30	66	264	876	0.27	2.0	20.4	16.63
19	F	1	49	30	33	695	876	0.08	1.0	20.4	8.31
20	F	2	52	30	21	982	876	0.04	0.6	23.8	6.97
21	F	2	49	30	41	679	876	0.09	1.2	20.4	10.33
22	F	3	49	30	66	409	876	0.16	2.0	20.4	16.63
23	F	3	49	30	41	391	876	0.1	1.2	20.4	10.33
24	F	4	52	30	21	1077	876	0.04	0.6	23.8	6.97
25	F	4	55	34	24	875	876	0.05	0.8	27.4	11.61
26	F	6	52	30	21	798	876	0.06	0.6	23.8	6.97
27	F	7	52	30	21	1294	876	0.05	0.6	23.8	6.97
28	F	8	52	30	21	1294	876	0.04	0.6	23.8	6.97
29	F	11	52	30	21	634	876	0.05	0.6	23.8	6.97
30	F	0	49	30	33	409	876	0.1	1.0	20.4	8.31
31	F	0	52	30	41	215	876	0.16	1.2	23.8	13.60
32	F	0	49	30	41	191	876	0.18	1.2	20.4	10.33
33	F	1	52	30	21	634	876	0.04	0.6	23.8	6.97
34	F	1	52	30	21	634	876	0.04	0.6	23.8	6.97
35	F	4	53	30	26	470	876	0.03	0.8	25.1	9.46
36	F	4	49	30	66	121	876	0.23	2.0	20.4	16.63
37	F	6	49	30	26	836	876	0.01	0.8	20.4	6.55
38	F	7	49	30	26	664	876	0.02	0.8	20.4	6.55
39	F	6	56	34	19	780	876	0.05	0.6	28.3	9.87
40	M	0	52	30	21	649	876	0.08	0.6	23.8	6.97
41	M	0	52	30	21	565	876	0.1	0.6	23.8	6.97
42	M	0	54	30	21	605	876	0.07	0.6	26.2	8.29
43	M	6	49	30	66	592	876	0.12	2.0	20.4	16.63
44	M	1	45	25	63	895	876	0.11	1.6	15.3	8.37
45	M	3	49	30	66	340	876	0.39	2.0	20.4	16.63
46	M	3	49	30	33	449	876	0.12	1.0	20.4	8.31
47	M	0	49	30	66	429	876	0.21	2.0	20.4	16.63
48	F	1	59	34	30	270	876	0.21	1.0	31.8	19.41
49	M	6	56	34	19	780	876	0.05	0.6	28.3	9.87
50	M	1	53	30	21	855	876	0.04	0.6	25.1	7.64
51	F	2	52	30	53	391	876	0.18	1.6	23.8	17.58
52	F	2	49	30	53	762	876	0.11	1.6	20.4	13.35
53	F	3	52	30	21	1294	876	0.05	0.6	23.8	6.97
54	F	8	52	30	21	836	876	0.04	0.6	22.9	6.69
55	F	9	49	30	66	365	876	0.41	2.0	20.4	16.63
56	F	17	49	30	66	297	876	0.22	2.0	20.4	16.63
57	F	19	49	30	33	605	876	0.07	1.0	20.4	8.31
58	F	20	49	30	53	409	876	0.1	1.6	20.4	13.35
59	F	30	54	30	26	875	876	0.07	0.8	26.2	10.26
60	M	1	49	30	66	183	876	0.32	2.0	20.4	16.63
61	M	3	49	30	66	340	876	0.39	2.0	20.4	16.63
62	M	3	49	30	33	449	876	0.12	1.0	20.4	8.31
63	F	0	58	34	30	297	876	0.25	1.0	30.7	18.09
64	F	1	53	30	53	383	876	0.18	1.6	25.1	19.29
65	M	5	49	30	66	382	876	0.12	2.0	20.4	16.63
66	M	5	52	30	53	310	876	0.22	1.6	23.8	17.58
67	M	4	52	30	21	960	876	0.05	0.6	23.8	6.97
68	M	0	52	30	21	836	876	0.07	0.6	23.8	6.97
69	M	9	52	30	26	836	876	0.06	0.8	23.8	8.62
70	F	0	49	30	66	391	876	0.2	2.0	20.4	16.63
71	M	0	59	33	30	270	876	0.22	1.0	31.8	18.84
72	F	14	49	30	66	290	876	0.46	2.0	20.4	16.63
73	F	1	42	25	49	1486	876	0.06	1.2	14.0	5.20
74	F	14	49	30	66	310	876	0.24	2.0	20.4	16.63
75	F	15	49	30	33	649	876	0.05	1.0	20.4	8.31
76	F	15	49	30	33	745	876	0.04	1.0	20.4	8.31
77	F	17	53	30	33	247	876	0.11	1.0	25.1	12.01
78	F	17	49	30	66	215	876	0.28	2.0	20.4	16.63
79	F	0	49	30	41	565	876	0.08	1.2	20.4	10.33
80	F	0	54	30	53	145	876	0.39	1.6	26.2	20.91
